# Can Arterial Blood Gas, Electrolyte and Acid–Base Analysis at Admission be Used to Predict Survival to Hospital Discharge for Different Causes of Colic?

**DOI:** 10.1002/vms3.70210

**Published:** 2025-02-06

**Authors:** Peter I. Milner, David Bardell

**Affiliations:** ^1^ Department of Equine Clinical Sciences Institute of Infection Veterinary and Ecological Sciences University of Liverpool Neston UK

**Keywords:** arterial blood analysis, colic, horse, prediction modelling

## Abstract

**Background:**

Predicting outcome in horses presenting with colic remains challenging.

**Objectives:**

To test whether arterial blood samples in horses admitted for colic predict outcome to hospital discharge for different colic types.

**Methods:**

Arterial blood samples collected on admission from 358 horses undergoing medical or surgical management of colic were evaluated for pH, PaO_2_, PaCO_2_, Na^+^, K^+^, iCa^2+^, Cl^−^, HCO_3_^−^ (P), HCO_3_^−^ (P, set), Base (B), Base (ecf) and anion gap. Categories were small intestinal non‐strangulating (SINS) or strangulating (SIS) lesions, large colon non‐volvulus (LCNV) or volvulus (LCV), small colon non‐strangulating (SCNS) or strangulating (SCS) lesions, viscus rupture or other. Multivariable logistic regression models were developed based on survival, or not, to hospital discharge. Odd ratios (ORs) with 95% confidence intervals (95%CI) and area under the curve receiver operator characteristics (AUROC), sensitivity, specificity and positive and negative predictive values were calculated at a cut‐off value of *p* = 0.5.

**Results:**

A total of 295/358 (82.4%) horses survived to hospital discharge. Variables retained as significantly associated with survival were PaO_2_ for SINS (OR 1.15, 95%CI 1.04–1.27), PaO_2_ (OR 1.06, 95% CI 1.01–1.11) and Na^+^ (OR 1.24, 95% CI 1.02–1.52) for SIS, Ca^2+^ (OR 175.1, 95% CI 2.20–13958) and HCO_3_^−^ (P) (OR 1.18, 95% CI 1.01–1.37) for LCNV and PaCO_2_ (OR 1.47, 95% CI 1.05–2.06) for LCV. AUROCs showed acceptable‐excellent discrimination (range: 0.7–0.9), excellent sensitivity (range: 91%–100%) but poor–fair specificity (range: 8%–50%).

**Conclusions:**

Arterial blood is good at predicting survival based on colic type but less accurate at predicting those horses which do not survive to hospital discharge.

## Graphical abstract

This study used arterial blood analysis taken on admission to determine which measures were associated with survival to hospital discharge in a cohort of horses presenting to a single clinic for medical and surgical management of colic. Specific arterial blood measures were found to be associated with survival in non‐strangulating and strangulating small intestinal and in large colon volvulus and non‐volvulus conditions

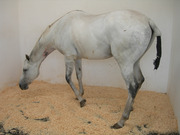

## 1 Introduction

Outcome in horses admitted for colic can be difficult to predict at initial presentation. Different causes of colic can be associated with distinct prognoses, for example survival to hospital discharge is reduced with a strangulating lesion compared to a simple obstruction (Mair and Smith 2005). Physical examination may be limited due to the size of the horse and venous blood samples may not truly reflect systemic alterations occurring during disease states (Gunes and Atalan 2006). Discrepancies have been reported between venous and arterial blood, particularly in relation to pH and pCO_2_ in haemodynamically unstable patients and hence arterial blood analysis may provide a more appropriate indication of circulatory compromise in critical patients (Archer 2019).

Clinical prediction models have been developed in human and veterinary medicine to enhance decision making and to estimate the risk of disease or its outcome based on prognostic or risk factors gathered at the time of examination. Several authors have investigated the utility of a single variable (e.g., peripheral blood lactate or packed cell volume), combination of multiple clinicopathological measurements to create multivariable models or scoring systems to predict survival in horses (Delesalle et al. 2007; Orsini et al. 1988; Reeves et al. 1989; Furr, Lessard, and White 1995). Recently we have described that components of arterial blood gas, electrolyte and acid–base analysis, particularly ionised calcium (iCa^2+^), are associated with the managing horses medically or surgically, as well as being implicated in the survival of colic cases, particularly those undergoing surgical management of small intestinal disease (Viterbo et al. 2023).

In the present study we investigated the hypothesis that arterial blood gas, electrolyte and acid–base analysis would be able to discriminate between horses which survive to hospital discharge from those which do not for different colic types. Our aim was to develop models to test how well arterial blood gas, electrolyte and acid–base analysis obtained at the time of admission to a single XXXX clinic for investigation of colic predicted the outcome for the horse depending on the colic type. To achieve this, we compared arterial blood gas, electrolyte and acid–base measures in horses admitted for colic and determined their outcome to hospital discharge based on final diagnosis.

## 2 Materials And Methods

### 2.1 Study Population

Horses presenting to XXXX for medical or surgical management of colic between June 2010 and December 2023 were enrolled in the study. Institutional ethical approval (XXXX) and owner consent (XXXX) were obtained for the study. Horses were included if they were > 1 year of age and had an arterial blood sample obtained at the time of admission to the clinic.

Cases were categorised based on diagnosis as small intestinal non‐strangulating (SINS) or strangulating lesions (SIS), large colon non‐volvulus (LCNV) or volvulus (LCV), small colon non‐strangulating (SCNS) or strangulating (SCS), viscus rupture or other. Final diagnosis was based on findings at surgical exploration or where post‐mortem was performed. For non‐surgical cases, diagnosis was based on clinical data gathered at admission and during hospital management and reflected the final diagnosis recorded by the senior clinician in the clinical records. Horses diagnosed with non‐gastrointestinal cause of colic (e.g., renal mass, uterine torsion) were excluded as were those where euthanasia was performed at admission due to financial constraints or a putative hopeless prognosis and no further investigations or treatment was instigated. Horses undergoing surgical management which died during surgery or during recovery from general anaesthesia were included in the analysis whereas cases which were euthanised intra‐operatively or because of a catastrophic injury during recovery from general anaesthesia were removed from analysis. Survival was defined as alive when discharged from the hospital.

### 2.2 Arterial Blood Gas Collection

Arterial blood samples were obtained by one operator (XX) and as described by Viterbo et al. (2023). Briefly, with the horse restrained in stocks and breathing ambient air, samples were collected via direct needle puncture from the common carotid artery anaerobically into pre‐heparinised syringes using a 21‐gauge 38 or 50 mm needle. Samples were analysed immediately using either an ABL77 (Radiometer Medical, Denmark) or Rapid Point 500 (Siemens, UK) blood gas analyser. Both systems utilised automatic calibration every 30–240 min (analyser‐dependent) and following installation of new cartridges.

Measured variables from the arterial blood samples included pH, partial pressure of oxygen (PaO_2_, mmHg), partial pressure of carbon dioxide (PaCO_2_, mmHg) and concentrations of sodium, (Na^+^; mmol L^−1^), potassium (K^+^; mmol L^−1^), ionised calcium (iCa^2+^; mmol L^−1^) and chloride (Cl^−^; mmol L^−1^). Actual and standardised plasma bicarbonate concentration (HCO_3_^−^ (P) and HCO_3_^−^ (P, st), respectively), blood and extracellular fluid base excess (Base (B) and Base (ecf), respectively) (all mmol L^−1^) and anion gap (mEq L^−1^) were calculated from pre‐programmed algorithms.

### 2.3 Data Analysis

Statistical analysis was performed using SPSS statistical software version 29 for Windows (IBM, Chicago, IL, USA). Univariable analysis of arterial blood gas, electrolyte and acid–base variables was performed for each colic‐type between horses that did or did not survive to hospital discharge. Distribution of data was assessed using visual inspection of histograms, Q–Q plots, Kolmogorov–Smirnov and Shapiro–Wilk tests for Normality and Levene's test for equality of variance. Unpaired student's *t*‐test or Mann–Whitney U test were used for univariable analysis. An adjusted *p*‐value of *p* < 0.05/10 = 0.005 was used for multiple comparisons using Bonferroni correction. Results are presented as mean (95% confidence intervals, CI) or median (interquartile range, IQR).

All variables were then screened for collinearity using Pearson's r or Spearman's rho and the most statistically significant or biologically plausible variable was selected where variables were highly correlated (> 0.9). Variables were entered into a forward likelihood ratio method to determine final multivariable logistic models related to survival to hospital discharge for each type of colic with outcome as survived/did not survive. Variables with univariable *p*‐values of < 0.2 were entered in a stepwise fashion into the models and retained if they significantly improved the fit (*p* < 0.05). Hosmer–Lemeshow goodness of fit test statistic was used to assess the fitness of each model. To measure each models’ ability to discriminate between the two outcomes (survived/did not survive), area under curve receiver operator characteristics (AUROC) values (95% CI) were calculated. Predictive outcomes (*p*) for each model were calculated using *p* = e^(^ *^x^* ^)^/1+e^(^ *^x^* ^)^ (where *x* = *β* + (*α*_1_*X_1_ + ….*α*_n_*X_n_) and *α* = coefficient of retained variable *X* and *β* = intercept of the model). Sensitivity, specificity and positive and negative predictive values were then calculated for each model using a cut‐off value of *p* = 0.5.

## 3 Results

**FIGURE 1:**
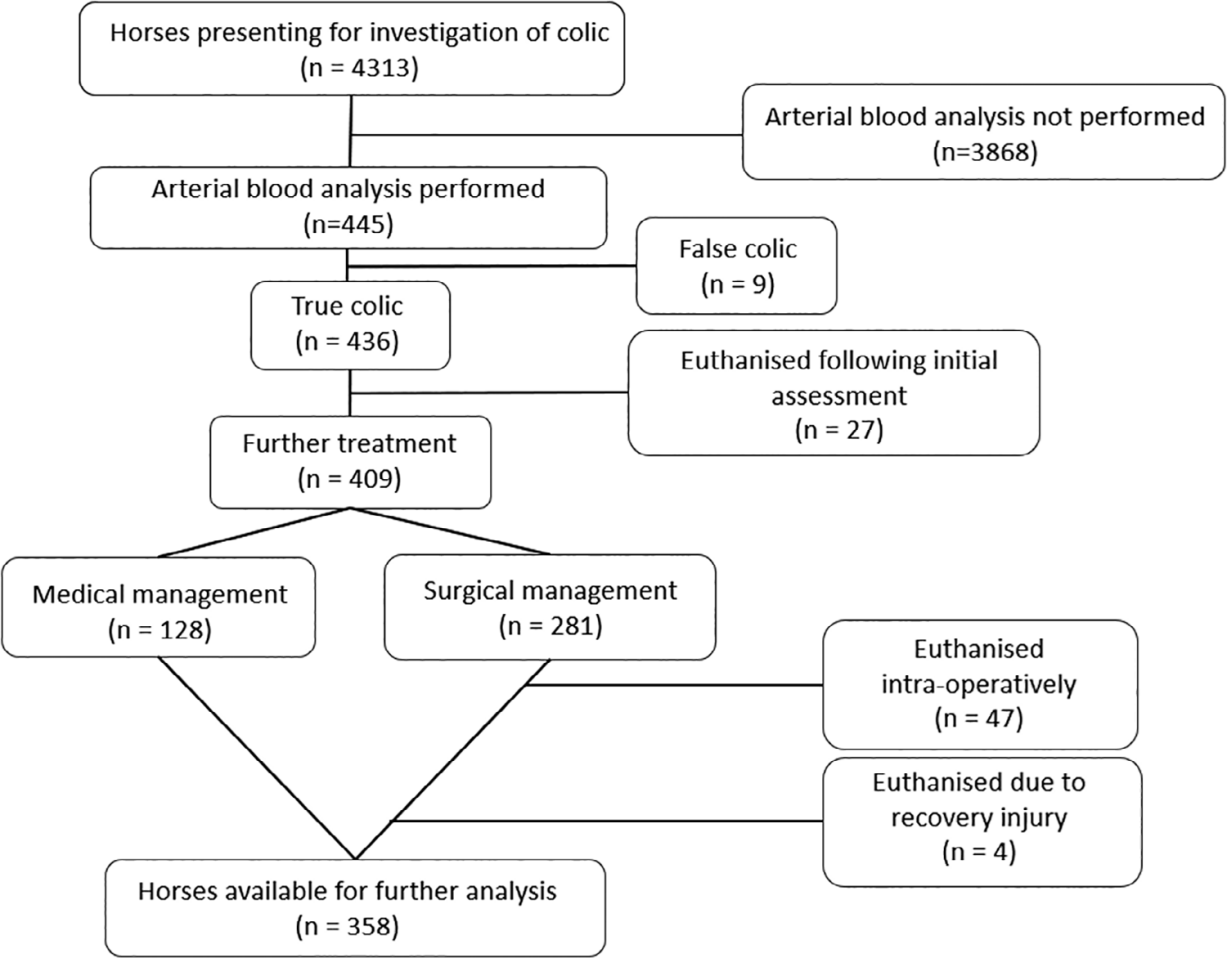
Flow chart illustrating reasons for the exclusion of horses presenting for colic to XXXX between June 2010 and December 2023 from the final analysis.

Over the study period, 4313 horses with suspected colic were presented to the clinic. Arterial blood gas analysis was performed on 445 horses on admission, of which 379 were included in a previous study (Viterbo et al. 2023). Non‐gastrointestinal causes of colic were identified in 9/445 horses and these were subsequently excluded from this study. Of the remaining 436 horses, 27 were euthanised on presentation due to economic or putative poor prognosis following initial examination, leaving 409 horses admitted for true colic which underwent further treatment. Following initial examination, 281 horses underwent exploratory laparotomy under general anaesthesia whereas 128 horses were treated medically. Of the 281 horses undergoing exploratory laparotomy, 47 were euthanised intra‐operatively due to economic and/or putative prognostic reasons (including all six horses with viscus rupture) and four following catastrophic injury during recovery leaving a total of 358 horses available for analysis (Figure 1). Lesion type for horses included in the final analysis was: SINS, *n* = 53 (15%); SIS, *n* = 99 (28%); LCNV, *n* = 140 (39.1%); LCV, *n* = 26 (7%); SCNS, *n* = 6; SCS, *n* = 4 and other, *n* = 30 (8%). Table S1 shows the distribution of colic‐types undergoing medical or surgical management. Out of 358 horses analysed, 295 (82.4%) survived to hospital discharge (survived: SINS, *n* = 41/53 (77%); SIS, *n* = 69/99 (70%); LCNV, *n* = 126/140 (90%); LCV, *n* = 20/26 (77%); SCNS, *n* = 6/6; SCS, *n* = 3/4; other, *n* = 30/30 (100%).

### 3.1 Arterial Blood Analysis for Survival to Hospital Discharge Based on Colic Diagnosis

Table 1 shows mean (95% CI) or median (IQR) of each measured variable from the arterial blood analysis for horses surviving to hospital discharge for SINS, SIS, LCNV and LCV categories of colic. There were insufficient numbers in the survived/did not survive to hospital discharge groups for SCNS, SCS or Other for meaningful statistical analysis. Following Bonferroni correction, only PaO_2_ remained significantly different in horses diagnosed with SINS between those horses that survived or did not survive to hospital discharge (Table 1).

**TABLE 1:** Univariable analysis of arterial blood gas, electrolyte and acid–base variables taken on admission in horses that survived or did not survive to hospital discharge. Diagnosis classified as small intestinal non‐strangulating lesion (SINS), small intestinal strangulating lesion (SIS), large colon non‐volvulus (LCNV) or large colon volvulus (LCV). Un‐paired *t* tests were used for normally distributed data, and Mann–Whitney U tests† were used for non‐normally distributed data, with significance assumed at *p* < 0.05. Variables retaining significance following Bonferroni correction (*p* < 0.05/10 = 0.005) are highlighted in **bold**. Data are presented as mean (95% CI) or median (IQR).

	SINS (*n* = 53)			SIS (*n* = 99)			LCNV (*n* = 140)			LCV (*n* = 26)		
	Survived (*n* = 41)	Did not survive (*n* = 12)	*p*‐value	Survived (*n* = 69)	Did not survive (*n* = 30)	*p*‐value	Survived (*n* = 126)	Did not survive (*n* = 14)	*p*‐value	Survived (*n* = 20)	Did not survive (*n* = 6)	*p*‐value
pH	7.42 (7.40; 7.44)	7.43 (7.40; 7.45)	0.7	7.44 (7.43; 7.45)	7.44 (7.40; 7.48)	0.8†	7.44 (7.43; 7.44)	7.44 (7.34; 7.54)	0.7†	7.43 (7.41; 7.45)	7.44 (7.14; 7.74)	0.7†
PaO_2_ (mmHg)	89.4 (86.8; 92.0)	81.2 (76.9; 85.5)	**0.003**	92.6 (90.1; 95.0)	86.7 (82.8; 90.5)	0.007	91.7 (89.9; 93.4)	92.7 (88.4; 97.0)	0.7	89.0 (85.2; 92.8)	92.8 (74.4; 111.3)	0.6
PaCO_2_ (mmHg)	41.2 (39.7; 46.6)	42.2 (39.3; 45.0)	0.5	42.5 (41.0; 44.0)	43.7 (41.5; 46.0)	0.5	41.6 (40.8; 42.3)	39.4 (35.4; 43.4)	0.3	44.0 (42.3; 45.6)	35.0 (26.7; 43.3)	0.04
Na^+^ (mmol L^−1^)	133.6 (132.7; 134.5)	132.3 (129.2; 135.3)	0.7	133.8 (133.2; 134.3)	132.6 (131.6; 133.6)	0.06†	135.0 (131.0; 139.0)	134.0 (129.0; 139.0)	0.3†	135.0 (131.0; 139.0	134.0 (114.0; 154.0)	0.7†
K^+^ (mmol L^−1^)	3.30 (3.17; 3.43)	3.33 (3.03; 3.64)	0.8	3.23 (3.13; 3.34)	3.08 (2.93; 3.23)	0.1†	3.37 (3.30; 3.44)	3.15 (2.82; 3.49)	0.1†	3.35 (3.11; 3.59)	4.04 (2.97; 5.11)	0.2
Ca^2+^ (mmol L^−1^)	1.45 (1.31; 1.56)	1.39 (1.32; 1.47)	0.3†	1.33 (1.30; 1.36)	1.32 (1.09; 1.55)	0.1†	1.47 (1.33; 1.61)	1.36 (1.28; 1.44)	0.02†	1.42 (1.36; 1.48)	1.35 (1.15; 1.54)	0.4†
Cl^−^ (mmol L^−1^)	98.5 (96.9; 100.1)	95.8 (93.2; 98.4)	0.1	97.5 (96.4; 98.5)	95.4 (93.5; 97.4)	0.06†	100.3 (99.4; 101.1)	97.9 (94.0; 101.7)	0.2†	100.3 (98.6; 102.0)	101.3 (89.1; 113.6)	0.4†
HCO_3_^−^ (P) (mmol L^−1^)	26.4 (25.2; 27.6)	27.4 (25.2; 29.5)	0.5	28.1 (27.0; 29.3)	29.0 (27.0; 30.9)	0.5	27.5 (26.9; 28.0)	25.8 (22.5; 29.0)	0.4†	28.5 (27.1; 29.9)	21.2 (13.5; 28.9)	0.02†
HCO_3_^−^ (P, st) (mmol L^−1^)	26.3 (25.3; 27.4)	27.0 (25.2; 28.9)	0.5	27.9 (26.9; 28.9)	28.6 (26.9; 30.2)	0.5	27.4 (26.9; 27.9)	26.3 (23.7; 28.8)	0.6†	28.0 (26.7; 29.3)	27.2 (22.1; 32.3)	0.06†
Base (B) (mmol L^−1^)	2.00 (0.80; 3.21)	2.85 (0.78; 4.20)	0.5	3.71 (2.61; 4.80)	4.39 (2.62; 6.16)	0.6	3.17 (2.63; 3.72)	2.90 (‐2.50; 5.40)	0.6†	3.90 (2.39; 5.40)	3.05 (‐2.85; 8.95)	0.05†

**FIGURE 2:**
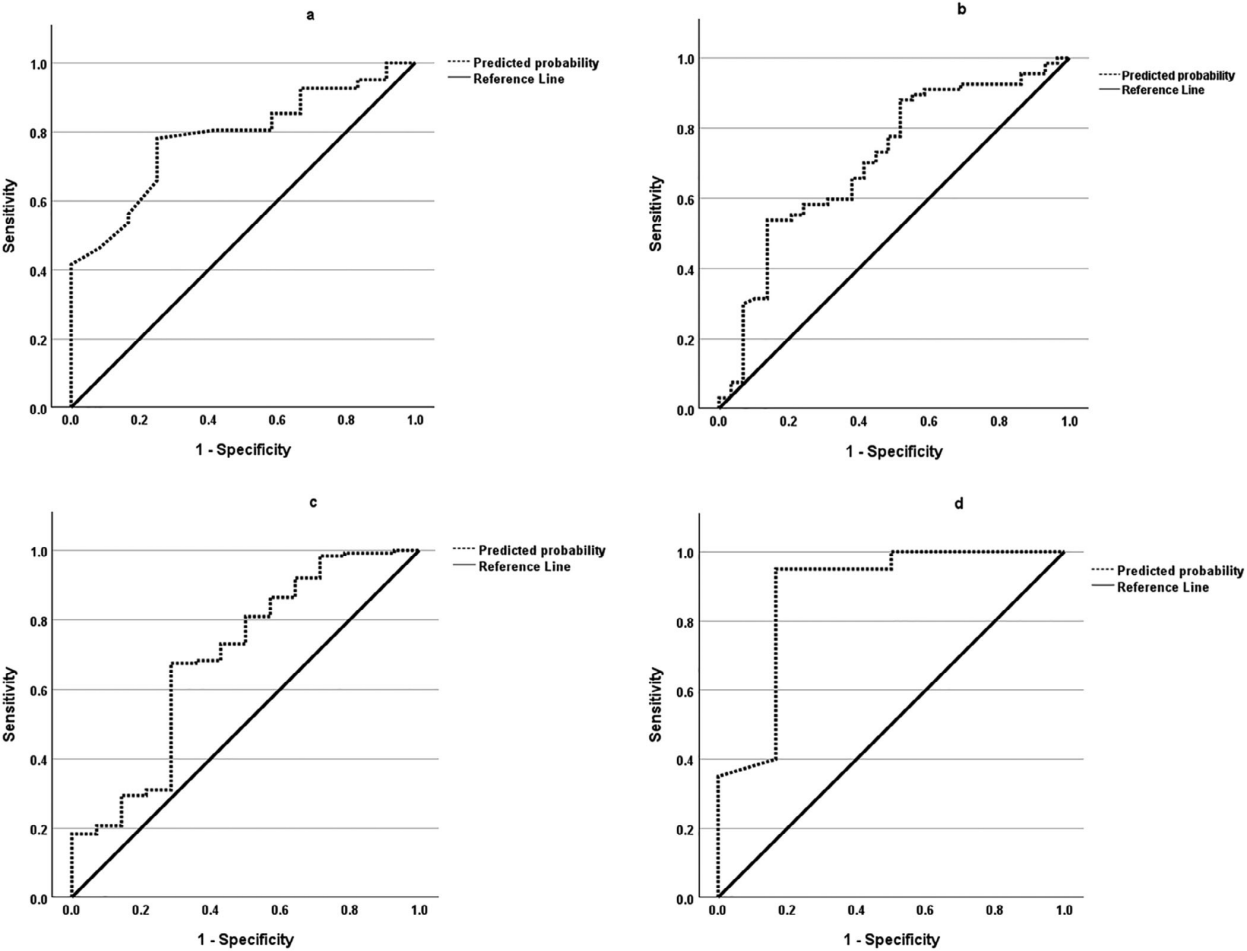
Receiver operating curves (ROC) for Models 1–4 where (a) Model 1 relates to horses diagnosed with small intestinal non‐strangulating lesions, (b) Model 2 relates to horses diagnosed with small intestinal strangulating lesions, (c) Model 3 relates to horses diagnosed with large colon non‐volvulus and (d) Model 4 relates to horses diagnosed with large colon volvulus.

Prediction models for SINS, SIS, LCNV and LCV were developed to explore the outcome of the horse with arterial blood gas, electrolyte and acid–base variables obtained on admission. Collinearity between HCO_3_^−^ (P), HCO_3_^−^ (P, st), Base (B) and Base (ecf) was high (> 0.9) so HCO_3_^−^ (P) was chosen as the most plausible variable and retained for each model. PaO_2_ was retained in Model 1 (SINS) (Table 2). The AUROC for Model 1 was 0.8 (95% CI 0.7; 0.9) (Figure 2a). The sensitivity and specificity of Model 1 was 92.7% and 33.3%, respectively. PPV was 82.6% and NPV was 57.1%. PaO_2_ was also retained for Model 2 (SIS) as well as Na^+^ (Table 3). The AUROC for Model 2 was 0.7 (95% CI 0.6; 0.8) (Figure 2b) with a sensitivity and specificity of 91% and 31%, respectively. PPV for Model 2 was 75% and NPV was 60%. Ca^2+^ and HCO_3_^−^ (P) were retained in Model 3 (LCNV) (Table 4). The AUROC for Model 3 was 0.7 (95% CI 0.5; 0.9) (Figure 2c). Sensitivity and specificity for Model 3 were 100% and 7.7%, respectively. PPV was 91.2% and NPV was 100%. Finally, PaCO_2_ was the only variable retained for Model 4 (LCV) (Table 5). The AUROC for Model 4 was 0.9 (95% CI 0.7; 1.1) (Figure 2d). Sensitivity and specificity of Model 4 were 95% and 50%, respectively. PPV was 86.3% and NPV was 75%.

**TABLE 2:** Multivariable regression model^a^ for survival to discharge in horses with small intestinal non‐strangulating lesion (*n* = 53).

Variable	Coefficient	Standard error	Adjusted odds ratio	95% Confidence interval	Likelihood ratio *p*‐value
PaO_2_	0.14	0.05	1.15	(1.04; 1.27)	0.007
Intercept	−10.65	4.35			

^a^ Multivariable logistic regression equation, *x* = −10.65 + (0.14 × PaO_2_), where predicted probability (*p*) = e*^x^*/(1 + e*^x^*).

**TABLE 3:** Multivariable regression model^a^ for survival to discharge in horses with small intestinal strangulating lesion (*n* = 96).

Variable	Coefficient	Standard error	Adjusted odds ratio	95% Confidence interval	Likelihood patio *p*‐value
PaO_2_	0.06	0.02	1.06	(1.01; 1.11)	0.02
Na^+^	0.22	0.10	1.24	(1.02; 1.52)	0.03
Intercept	−33.40	14.1			

^a^Multivariable logistic regression equation, *x* = −33.40 + [(0.06 × PaO_2_) + (0.22 × Na^+^)], where predicted probability (*p*) = e*^x^*/(1 + e*^x^*).

**TABLE 4:** Multivariable regression model^a^ for survival to discharge in horses with large colon non‐volvulus (*n* = 137).

Variable	Coefficient	Standard error	Adjusted odds ratio	95% Confidence interval	Likelihood ratio *p*‐value
iCa^2+^	5.17	2.23	175.1	(2.20; 13957.7)	0.02
HCO_3_^−^ (P)	0.16	0.08	1.18	(1.01; 1.37)	0.03
Intercept	−9.43	4.01			

^a^Multivariable logistic regression equation, *x* = −9.43 + [(5.17 × iCa^2+^) + (0.16 × HCO_3_^−^ (P))], where predicted probability (*p*) = e*^x^*/(1 + e*^x^*).

**TABLE 5:** Multivariable regression model^a^ for survival to discharge in horses with large colon volvulus (*n* = 26).

Variable	Coefficient	Standard error	Adjusted odds ratio	95% Confidence interval	Likelihood ratio *p*‐value
PaCO_2_	0.38	0.17	1.47	(1.05; 2.06)	0.03
Intercept	−14.40	6.90			

^a^ Multivariable logistic regression equation, *x* = −14.40 + (0.38 × PaCO_2_), where predicted probability (*p*) = e*^x^*/(1 + e*^x^*).

## 4 Discussion

This study showed that arterial blood analysis performed at the time of hospital admission may provide additional information on likelihood of survival in horses presenting with small intestinal and large colon causes of colic and undergoing medical or surgical management. However, arterial blood analysis was less accurate in identifying those horses which did not survive to hospital discharge and we were unable to be develop models relating to conditions involving the small colon or other cases where the colic was unrelated to a small intestinal or large colon condition. Where models were generated for horses presenting with small intestinal or large colon causes of colic, particular arterial blood variables were associated with survival related to specific categories of diagnosis. Of note we found that PaO_2_ was associated with survival to hospital discharge in those horses with small intestinal non‐strangulating disease and, alongside plasma Na^+^, PaO_2_ was also associated with survival in horses with strangulating small intestinal disease. For large colon non‐volvulus conditions, actual plasma bicarbonate (HCO_3_^−^ (P)) with iCa^2+^ was associated with survival whereas in horses with large colon volvulus, partial pressure of carbon dioxide (PaCO_2_) was clearly associated with survival to hospital discharge. This builds on our previous work where arterial blood analysis was used to establish differences in horses with colic compared to non‐colic cases and in particular, differentiating between different types of colic.^8^

In the present study we used arterial blood samples collected at the time of admission. Arterial blood is a more sensitive indicator than venous blood in the patient with circulatory compromise when compared to the normal stable patient (Gunes and Atalan 2006; Archer 2019; Chong, Saha, and Medarov 2021). Although we recognise that we did not directly compare arterial to venous blood in our present study, arterial blood analysis may be more relevant to use in horses with gastrointestinal disease where haemodynamic stability is often compromised (Collatos, Barton, and Moore 1995; Fogle et al. 2017). Standardising sampling is important for any investigation. In this study, one operator was responsible for sample collection (thereby reducing inter‐operator error) and samples were taken as soon as was feasible after admission and prior to any pharmacological interventions, although it is to be recognised that prior administration of pharmaceuticals may have occurred but could not be controlled for.

Cases included in the final analysis had a small intestinal or large colon cause of colic. Unfortunately, due to insufficient numbers, we were unable to develop models relating to conditions involving the small colon or other cases where the colic was unrelated to the small intestine or large colon. Cases where the horse died during surgery or in the period during recovery from general anaesthesia were included in the final analysis since death would likely be related to underlying condition whereas those cases which were euthanised due to external influences such as economics or putative prognosis were excluded. We chose to exclude the latter cases to minimise the effect of bias on outcome from non‐biological influences such as finances, management concerns from owners/caretakers (e.g., behavioural issues, co‐morbidities) or the effect of different surgeons who may advise to continue, or not with surgery based on personal experience. We also chose to define our diagnoses categories based on location as well as type of insult, whereas recent work by Long et al. (2024) grouped together all causes of strangulating obstruction (small and large intestine) thereby assuming that lesion behaviour was similar irrespective of location (Long et al. 2024). Categorising cases based on medical or surgical management rather than colic type may seem logical since some cases such as strangulating lesions are inherently managed surgically. However we found that some non‐strangulating lesions (e.g., small intestinal impactions), which could have been managed medically, underwent exploratory laparotomy primarily to rule out a possible strangulating lesion. Therefore, categorising cases based on the decision made for surgical or non‐surgical management is not clear cut and we believed that lesion distribution and subsequent survival (or not) would be better linked to colic type.

All four models generated in our study (Model 1: SINS; Model 2: SIS; Model 3: LCNV; Model 4: LCV) had acceptable to excellent discrimination (AUROC 0.7–0.9). Sensitivity for each model was generally excellent (91%–100%) suggesting each model provided a high degree of accuracy of being able to identify those horses within the cohort which survived to hospital discharge given the diagnosis. However, the models were generally less accurate at identifying those horses which did not survive to hospital discharge (specificity 8%–50%). The sensitivity and specificity of the models were generated using cut‐off values of *p* = 0.5 with the assumption that predictive probability values of 0.5 or less mean the horse would not survive and above 0.5 that the horse would survive to discharge. The positive and negative predictive values for each model were generally good to excellent (apart from NPV for Model 1). Models with high PPVs and NPVs should be good at predicting the correct outcome. In the present study this means that for each model tested, those horses with that particular colic‐type would be correctly predicted by that model to survive to hospital discharge (high PPV) whereas (Model 1 apart), models with a high NPV would likely correctly predict those horses which would not survive to hospital discharge with a particular colic‐type.

We found that PaO_2_ on admission was associated with survival to hospital discharge in horses diagnosed with small intestinal disease. In those horses diagnosed with a non‐strangulating small intestinal lesion it was the sole variable retained both in univariable (after correction) and multivariable analyses. Hypoxaemia is not uncommon in horses undergoing exploratory laparotomy under general anaesthesia and likely to be exacerbated in the horse presenting with lowered PaO_2_ prior to induction (Hovda, Love, and Chiavaccini 2022). In our study PaO_2_ was treated as a continuous variable, unlike other studies where cut‐off values are pre‐determined (Hovda, Love, and Chiavaccini 2022; Espinosa et al. 2017) and therefore using Model 1, predicted probabilities (*p*) can be calculated based on actual values. For example, a horse diagnosed with a small intestinal non‐strangulating lesion and with a PaO_2_ on admission of 67 mmHg has a predicted probability for survival of *p* = 0.22 compared to a horse presenting with PaO_2_ of 93 mmHg which has a value of *p* = 0.91. Plasma Na^+^ was retained alongside PaO_2_ in Model 2 suggesting that circulating sodium levels on admission are implicated in survival to hospital discharge in cases of small intestinal strangulating lesion. In horses, hyponatraemia may result where there is loss, usually through diarrhoea (Stewart et al. 1995) but has also been reported in myositis and colic, (Divers et al. 1987) systemic inflammatory response syndrome (Migliorisi et al. 2022) and due to fluid sequestration in intestinal obstruction. (Adrogué and Madias 2000) The inclusion of plasma Na^+^ in Model 2 may therefore reflect greater systemic derangements occurring with small intestinal strangulating lesions. As an example, comparing two horses with a strangulating small intestinal lesion, both with PaO_2_ of 89 mmHg on admission but with different plasma Na^+^ levels (138 mmol L^−1^ in the first horse and 128 mmol L^−1^ in the second), the predicted probability for the first horse to survive to hospital discharge would be *p* = 0.91 whereas for the second horse it would be *p* = 0.52 suggesting a moderate effect of plasma Na^+^ on survival. Lower plasma Na^+^ may lead to cellular dysfunction through cell swelling and alterations to membrane transport systems which use sodium, such as the Na^+^/H^+^ (NHE) exchanger (Gagnon and Delpire 2021).

For large colon conditions, elements of bicarbonate regulation (HCO_3_^−^ (P) and PaCO_2_ featured as retained variables, alongside plasma iCa^2+^ for horses diagnosed with large colon non‐volvulus (LCNV). Systemic acid–base disturbances, such as reduced plasma HCO_3_^−^ can affect intestinal transport mechanisms, particularly related to ion regulation and intracellular pH (pH_i_), and this has been described in the colon (Charney and Haskell 1984). Reduction in arterial plasma bicarbonate levels is accompanied by decreased HCO_3_^−^ secretion by colonic mucosal cells, to maintain the blood‐lumen HCO_3_^−^ concentration gradient. This may occur independently of Na^+^/H^+^ and Cl^−^/HCO_3_^−^ exchange processes, but impact on their activity due to changes in pHi. Indeed, HCO_3_^−^ secretion by colonic mucosal cells is correlated to net Na^+^ absorption (Charney and Haskell 1984; Wagner, Kurtin, and Charney 1985) and thereby impacts on Na^+^‐linked transport mechanisms, such as glucose transport into enterocytes (Chen, Tuo, and Dong 2016). In a similar way, iCa^2+^ plays a critical role in ion‐transport, as well as many other physiological activities. Regulation of glucose absorption via intracellular calcium levels acting through activation of protein kinases (e.g., protein kinase C and MAP‐kinase pathways) (Helliwell, Rumsby, and Kellet 2003) will depend on calcium influx via epithelial Ca^2+^ channels, voltage‐operated calcium channels and store‐operated calcium channels. Alterations in nutrient transport into enterocytes by acid–base and calcium disturbances may affect enterocyte health and thereby provides a mechanism for how these variables may interact to influence the outcome of the animal.

In horses diagnosed with large colon volvulus (LCV), PaCO_2_ was the only retained variable related to outcome in Model 4. Initially in the univariable analysis, PaCO_2_ was significantly associated with horses which did not survive to hospital discharge (Table 1) but was not retained after Bonferroni correction. However, in the multivariable analysis it was retained in the final model, with the model showing strong discrimination for survived/did not survive (AUROC = 0.9). Hypocapnia may be present due to increased respiratory rate caused by restricted ventilation capacity in standing horses with a painful, distended large colon, but may also be associated with increases in systemic lactate levels and reduced bicarbonate levels (Eichenholz et al. 1962). Interestingly reduced HCO_3_^−^ (P) levels were recorded in large colon volvulus cases not surviving to hospital discharge in the univariable analysis although following Bonferroni correction this variable was no longer significant and was also not retained in the final model. Periods of hypocapnia lead to reduction in cardiac output and reduction in mucosal and serosal blood flow with concomitant serosal hypoxia (Gurzman and Kruse 1999) potentially perpetuating haemodynamic dysfunction and colon hypoperfusion in horses with colon volvulus. The effect of PaCO_2_ can be demonstrated with predicted probabilities using Model 4. For example, a horse diagnosed with large colon volvulus presenting with PaCO_2_ of 32 mmHg has a predicted probability of survival to hospital discharge of 0.11 compared to 0.93 when PaCO_2_ on admission is 44 mmHg. In the study by Kelleher et al. (2013) hypercapnia (PaCO_2_ >70 mmHg), presented as a categorical variable, was a negative predictor of survival to hospital discharge in horses with large colon volvulus, however, these measurements were obtained under general anaesthesia rather than in the standing horse and may not be directly comparable due to the influence of obtunded ventilation on PaCO_2_ in the anaesthetised horse.

One of the main limitations for our study was that we were not able to develop models relating to all colic‐types, particularly those involving conditions of the small colon, due to low numbers encountered through convenience sampling. This may have been due to only 10% of the total number of horses presenting for colic undergoing arterial blood analysis during the study period (due to using a single operator for sampling). In addition, in some colic types, where there was high survival to hospital discharge (e.g., 90% survival in horses with LCNV), the ability to correctly identify (discriminate) those horses not‐surviving was reduced, hence leading to low specificities in some models. Additional concerns would be the use of two different analysers over the study period. Although agreement between the two systems was confirmed in a previous study (Viterbo et al. 2023), neither machine has been specifically validated for use with equine blood.

In conclusion, although our models were less accurate in identifying those horses which do not survive to hospital discharge, we consider that analysis of arterial blood could make a valuable contribution to predicting survival to hospital discharge for horses diagnosed with small intestinal and large colon causes of colic. Predictive values generated from the models developed in this study could be used to inform owners/caretakers of horses as well as clinicians of the likely outcome during the management of certain types of colic.

## Author Contributions

All authors (P.I.M., D.B.) contributed to the design and execution of the study, drafting the manuscript, revisions and agreed to the final content and as such as accountable for its accuracy and content.

## Ethics Statement

The authors confirm that the ethics policies of the journal, as noted by the journal's author guideline page, have been adhered to and project approval via institutional ethical review (VREC1422; approval date 07/11/0203) has been received.

## Conflicts of Interest

The authors declare no conflicts of interest.

### Peer Review

The peer review history for this article is available at https://publons.com/publon/10.1002/vms3.70210.

## Supporting information



Supporting information

## Data Availability

The data that support the findings of this study are openly available in “Arterial blood gas, electrolyte and acid–base analysis at admission can be used to predict survival to hospital discharge in small intestinal and large colon causes of colic” at http://doi.org/10.17638/datacat.liverpool.ac.uk/2643.
