# Supplementary material for: Can Arterial Blood Gas, Electrolyte and Acid–Base Analysis at Admission be Used to Predict Survival to Hospital Discharge for Different Causes of Colic?
Source: Vet Med Sci. 2025 Feb 6;11(2):e70210. doi: 10.1002/vms3.70210 (PMC11800372; doi:10.1002/vms3.70210)
Supplement: Supplementary file 1 — Supporting information [file VMS3-11-e70210-s001.docx]

**Table S1.** Distribution of colic-types undergoing medical or surgical management (n=358 horses).

|  | SINS  (n=53) | SIS  (n=99) | LCNV  (n=140) | LCV  (n=26) | SCNS  (n=6) | SCS  (n=4) | Other*  (n=30) |
| --- | --- | --- | --- | --- | --- | --- | --- |
| Medical | 6 | 0 | 89 | 0 | 4 | 0 | 30 |
| Surgical | 47 | 99 | 51 | 26 | 2 | 4 | 0 |

*SINS = small intestinal non-strangulating lesion; SIS = small intestinal strangulating lesion; LCNV = large colon non-volvulus; LCV = large colon volvulus; SCNS = small colon non-strangulating lesion; SCS = small colon strangulating lesion.*

**Other = peritonitis (n=5); parasitism (n=4); spasmodic colic (n=2); gastric impaction (n=2); Grade 1 rectal tear (n=1) and nothing abnormal detected (n=15).*
